# Cardio‐Metabolic Risk Profile of Women With Endometriosis: A Population‐Based Study

**DOI:** 10.1002/edm2.70008

**Published:** 2024-10-13

**Authors:** Marzieh Saei Ghare Naz, Mahsa Noroozzadeh, Shahla Noori Ardebili, Maryam Mousavi, Fereidoun Azizi, Fahimeh Ramezani Tehrani

**Affiliations:** ^1^ Reproductive Endocrinology Research Center, Research Institute for Endocrine Sciences Shahid Beheshti University of Medical Sciences Tehran Iran; ^2^ Endocrine Research Center, Research Institute for Endocrine Sciences Shahid Beheshti University of Medical Sciences Tehran Iran; ^3^ The Foundation for Research & Education Excellence Vestavia Hills AL USA

**Keywords:** diabetes, endometriosis, hypertension, metabolic syndrome, Tehran Lipid and Glucose Study

## Abstract

**Aims:**

Endometriosis (EM) and metabolic disorders are frequent health problems among reproductive‐aged women worldwide. Cardio‐metabolic risk profile of women with EM is not well understood. We aimed to investigate the cardio‐metabolic risk profile of Iranian reproductive‐aged women with EM.

**Methods:**

This study included 976 female participants aged 20–45 years of Tehran Lipid and Glucose Study. Endometriosis was diagnosed based on the participants' self‐reported previous diagnosis of EM, which was confirmed by reviewing the relevant medical documentation. All biochemical measures (low‐density lipoprotein cholesterol [LDL], high‐density lipoprotein cholesterol [HDL], triglycerides [TG], and fasting blood glucose concentrations [FBG]) and measurement of systolic blood pressure (SBP) and diastolic blood pressure (DBP) and anthropometric parameters were performed according to the standard protocol of TLGS. Logistic regression analysis was performed to estimate the odds ratio of cardio‐metabolic disease.

**Results:**

Of the 976 study participants, 161 individuals (16.5%) had a confirmed diagnosis of endometriosis. There were no significant differences in the median of metabolic parameters among women with and without endometriosis (*p* > 0.05). The prevalence of metabolic syndrome was significantly higher in women with EM group compared to the non‐EM group (21.9% vs. 14.9%). The presence of endometriosis was associated with an increased odds of metabolic syndrome (adjusted odds ratio 1.99 [95% CI 1.20–3.30]; *p* = 0.007). And endometriosis significantly increased odds of low HDL by 2.07 (1.02–4.20); after adjustment, it still remained significant (*p* = 0.03). Endometriosis also increased odds of high waist circumstance significantly (1.58 [1.06–2.37]; *p* = 0.02).

**Conclusions:**

Women with endometriosis may be at an increased risk of developing metabolic syndrome, high waist circumstance and low HDL compared to their counterparts without the condition. Given the potential cardio‐metabolic implications, healthcare providers should consider assessing the metabolic profile of women diagnosed with endometriosis.

## Graphical abstract

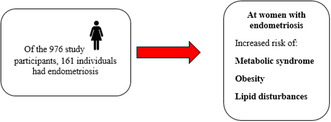

## 1 Introduction

Endometriosis (EM) is a common debilitating clinical condition among women that affects about 18% of them [1]. The clinical presentation of EM is not similar and patients may experience different symptoms including cyclic pelvic pain, menstrual dysfunction, dysmenorrhea, dyspareunia, ovulatory dysfunction and reproductive dysfunction [2]. Several factors have been identified as potential predisposing elements for the development of endometriosis, including alcohol intake, smoking, exposure to endocrine‐disrupting chemicals and early menarche. Moreover, prenatal exposure factors including being born prematurely, low birth weight, being formula‐fed and having been exposed to diethylstilbestrol (DES) in utero are known as predisposal factors for development of EM [3, 4, 5, 6]. Women with EM are more likely to experience obstetric complications such as miscarriage, preterm birth, small for gestational age, stillbirth, preeclampsia, placental abnormalities, haemorrhage in labour and caesarean section [7]. Women with EM are also at higher risk of experiencing major chronic diseases [8].

Meanwhile, women with endometriosis probably experience unfavourable metabolic parameters [9]. The underlying mechanisms linking endometriosis and metabolic dysregulation are not yet fully elucidated, but may involve shared inflammatory pathways, hormonal imbalances and lifestyle factors. A recent review supported that chronic inflammatory status in women with EM can cause metabolic disorder, cardiovascular diseases and immune dysregulation [10].

The Nurses' Health Study II demonstrated that women with endometriosis aged 25–42 years are at higher risk of hypercholesterolaemia and hypertension [11]; another publication based on data from this cohort reported that women aged 25–42 years with endometriosis did not exhibit an increased risk of developing type 2 diabetes compared to those without the condition [12]. A study among women with EM who participated in the National Health and Nutrition Examination Survey data showed that they suffer from lipid disturbances [9]. A study by Cirillo et al. [13] demonstrated that among women with mean age of 45 years total cholesterol, LDL‐C, triglycerides and homocysteinaemia, a high‐sensitive C‐reactive protein of women with stage III/IV of endometriosis were significantly higher than the control group. It is also reported that endometriosis can increase disorders such as stroke through hyperinflammatory milieu [14]. There is no recommendation about management of metabolic disorders among women with endometriosis in The European Society of Human Reproduction and Embryology (ESHRE) [15].

Despite the growing body of evidence suggesting an association between endometriosis and an unfavourable metabolic profile, the nature and extent of this relationship remain a subject of ongoing debate. The objective of this study was to compare the cardio‐metabolic risk profile, encompassing type 2 diabetes, metabolic syndrome, hypertension, pre‐diabetes, pre‐hypertension, obesity and dyslipidaemia among reproductive‐aged participants from the TLGS specifically distinguishing between those with and without endometriosis.

## 2 Methods

### 2.1 Study Design and Study Population

This population‐based study was conducted within the framework of the Tehran Lipid and Glucose Study (TLGS) that began in 1998 to investigate the prevalence, incidence, risk factors and characteristics of non‐communicable diseases (NCDs) in an urban population. The details of TLGS have been published elsewhere [16]. Data regarding demographic, anthropometric, reproductive, and metabolic traits, general medical examinations, and laboratory measures were collected, with follow‐up every 3 years. For the purposes of the present investigation, data collected during the 6th follow‐up visit of 976 premenopausal women aged 20–45 years were utilised. The endometriosis status of these participants was comprehensively ascertained using a standardised questionnaire and review of relevant medical documentation [17]. All participants were residents of urban areas.

### 2.2 Definition, Clinical and Laboratory Measurements

A trained staff collected related data (demographic, medical history and reproductive history) through interviews with participants and a general practitioner was assessed for the anthropometric and physical examinations [16]. Weight and height measurements were performed using a digital scale with an accuracy of 100 g and a stadiometer with an accuracy of nearest 0.5 cm. Body mass index (BMI) was calculated by dividing your weight in kilograms by square of height (meters). Physical activity was measured using a modifiable activity questionnaire (MAQ) and its levels were expressed as metabolic equivalent hours per week (METs h/week) [18, 19]. All biochemical measures (low‐density lipoprotein cholesterol [LDL], high‐density lipoprotein cholesterol [HDL], triglycerides [TG], fasting blood glucose concentrations [FBG]) and measurement of systolic blood pressure (SBP) and diastolic blood pressure (DBP) were performed according to the standard protocol of TLGS, which has been addressed elsewhere [16].

Type 2 diabetes (T2D) was defined as FBG ≥ 126 mg/dL or 2‐h plasma glucose concentrations ≥ 200 mg/dL, or treatment with antidiabetic medications. Pre‐diabetes is defined as fasting blood glucose or fasting plasma glucose of 100 mg/dL (5.6 mmol/L) to 125 mg/dL (6.9 mmol/L) or impaired fasting glucose or 2‐h PG of 140 mg/dL (7.8 mmol/L) to 199 mg/dL (11.0 mmol/L) (impaired glucose tolerance) in the 75‐g oral glucose tolerance test or drug treatment [20]. We defined pre‐hypertension prehypertension: SBP 120–139 mmHg and/or DBP 80–89 mmHg, and hypertension (SBP ≥ 140 mmHg or DBP ≥ 90 mmHg or taking antihypertensive drugs) [21]. Dyslipidaemia was defined as TC ≥ 240 mg/dL, TG > 200 mg/dL, LDL ≥ 160 mg/dL, HDL < 40 mg/dL and/or using lipid‐lowering drugs [22].

Participants who met three or more of the following criteria were diagnosed with metabolic syndrome: waist circumference (WC) ≥ 90 cm [23]; (2) systolic blood pressure (SBP) ≥ 130 mmHg or diastolic blood pressure (DBP) ≥ 85 mmHg or taking hypertension medications; (3) triglyceride (TG) level ≥ 150 mg/dL (1.70 mmol/L); (4) high‐density lipoprotein cholesterol (HDL‐C) < 50 mg/dL (1.3 mmol/L) or (5) fasting plasma glucose (FPG) level ≥ 100 mg/dL (5.6 mmol/L) or taking diabetes medications (1).

Women were requested to report whether they had been diagnosed with endometriosis. The medical records of those who reported such a diagnosis were subsequently verified. Individuals who reported mild to moderate chronic pelvic pain, dysmenorrhea or dyspareunia without a definitive endometriosis diagnosis were excluded from the analysis. The self‐reported endometriosis diagnosis has been validated in a previous study [24].

The ethics committee of the Research Institute for Endocrine Sciences, Shahid Beheshti University of Medical Sciences approved the study protocol. All participants signed the informed written consent form. The study was performed in adherence with the Declaration of Helsinki.

### 2.3 Statistical Analysis

Data were analysed using IBM SPSS Statistics version 22.0. Continuous variables are shown as means and SD or median (Q1–Q3). The normality of the distribution of variables was assessed using the Kolmogorov–Smirnov test, prior to analysis. The *T*‐student and Mann–Whitney *U* tests were used for continuous variables with or without normal distribution, respectively. Also, we used Chi‐squared test or Fisher exact test to compare categorical variables.

The *χ* ^2^ or Fisher exact tests were used for categorical variables. MetS, DM, pre‐DM, HTN, pre‐HTN, and each component of MetS also was assessed separately as response variables. Multiple logistic regression analysis was conducted to estimate the odds ratio of cardio‐metabolic disorders. Model 1 represented the crude effect of endometriosis on each pre‐specified cardio‐metabolic event. Model 2 included the adjustments made in Model 1, further accounting for variables including age, education, smoking and physical activity. Statistical significance was set at *p*‐value < 0.05.

## 3 Results

Among the 976 study participants, 161 (16.5%) had endometriosis. Demographic, anthropometric and clinical characteristics of the study participants are shown in Table 1. The median (Q1–Q3) age of the women with and without endometriosis was 36 (29.75–40) and 37 (32–42) years, respectively. There were no significant differences of median of metabolic parameters including FBS, LDL, HDL, TG cholesterol, among both women with and without endometriosis (*p* > 0.05).

**TABLE 1:** Demographic, anthropometric and clinical characteristics of the study participants according to the endometriosis status.

Variables		Endometriosis (*n* = 161)	Non‐endometriosis (*n* = 815)	*p*
Age (year)		36 (29.75–40)	37 (32–42)	0.005
Weight (kg)		67 (57–79)	66 (59–75)	0.50
BMI (kg/m^2^)		26.09 (22.76–30.46)	25.61 (22.94–28.81)	0.37
Hip circumferences (cm)		102 (96–110)	103 (98–108)	0.52
Physical activity		45 (28%)	282 (35.2%)	0.07
Ever smoker		5 (3.2%)	46 (6%)	0.16
Education	Illiterate	0	2 (0.2%)	0.42
	< 12 years	66 (41.2%)	300 (36.8%)	
	12 years	6 (3.8%)	20 (2.5%)	
	> 12 years	88 (55%)	493 (60.5%)	
FBS (mg/dL)		90 (85–96.25)	90 (85–96)	0.36
LDL (mg/dL)		100 (82.4–117.2)	102.2 (85.2–119.6)	0.22
HDL (mg/dL)		50 (45–56)	50 (43–58)	0.23
TG (mg/dL)		95 (69–136.75)	95.5 (67–136)	0.87
Cholesterol (mg/dL)		171 (149–193)	175 (154–197)	0.23
HDL/LDL		0.495 (0.389–0.616)	0.488 (0.396–0.615)	0.88
HDL/TC		0.288 (0.241–0.34)	0.29 (0.244–0.346)	0.90
SBP (mmHg)		101 (95–110)	101 (95–110)	0.76
DBP (mmHg)		70 (65–78)	70 (65–75)	0.92
T2DM		29 (18%)	114 (14.1%)	0.19
Pre‐T2DM		54 (33.5%)	236 (29%)	0.25
Metabolic syndrome		35 (21.9%)	120 (14.9%)	0.02
High waist		82 (50.9%)	370 (45.5%)	0.203
Dyslipidaemia		737 (90.5%)	151 (93.8%)	0.18
Decreased HDL		152 (94.4%)	725 (89.1%)	0.03
Elevated TG		66 (41%)	312 (38.3%)	0.52
Elevated FBS		44 (27.3%)	172 (21.1%)	0.08
Pre‐HTN		29 (18.8%)	110 (14.1%)	0.13
HTN		7 (4.3%)	34 (4.2%)	0.92

Abbreviations: BMI, body mass index; DBP, diastolic blood pressure; FBS, fasting blood glucose; HDL‐C, high‐density lipoprotein‐cholesterol; HTN, hypertension; SBP, systolic blood pressure; TG, triglycerides.

The prevalence of MetS and low HDL levels was significantly higher in the women with EM group compared to the non‐EM group (21.9% vs. 14.9%) and (94.4% vs. 89.1%), respectively. However, the prevalence of pre‐HTN, HTN, pre‐DM, and DM, dyslipidaemia, elevated FBS and TG were not significantly different between two groups (Table 1).

The presence of endometriosis in women was associated with an increased odds of metabolic syndrome (1.26 [1.02–1.56]; *p* = 0.02); the results remained statistically significant after adjusting for age, education, smoking and physical activity (adjusted odds ratio 1.99 [95% CI 1.20–3.30]; *p* = 0.007). However, in both unadjusted and adjusted models, endometriosis was not associated with an increased odds of diabetes (adjusted: 1.58 [0.93–2.67]), pre‐diabetes (adjusted: 1.03 [0.68–1.54]), hypertension (adjusted: 1.07 [0.43–2.69]), pre‐hypertension (adjusted: 1.41 [0.84–2.34]), dyslipidaemia (adjusted: 1.49 [0.69–3.22]) and elevated FBS (adjusted: 1.29 [0.83–1.98]). And endometriosis significantly increased odds of low HDL by 2.07 (1.02–4.20); after adjustment, it still remained significant (*p* = 0.03). And after adjustment, endometriosis increased the odds of high waist circumstance significantly (1.58 [1.06–2.37]; *p* = 0.02) (Table 2).

**TABLE 2:** Odds ratios (95% CI) of endometriosis on every cardio‐metabolic event.

Response variables	Model 1		Model 2	
	Odds ratio (95% confidence interval)	*p*	Odds ratio (95% confidence interval)	*p*
Metabolic syndrome	1.26 (1.02–1.56)	0.02	1.99 (1.20–3.30)	0.007
Hypertension	1.04 (0.45–2.38)	0.92	1.07 (0.43–2.69)	0.87
Pre‐hypertension	1.40 (0.89–2.21)	0.13	1.41 (0.84–2.34)	0.18
Pre‐diabetes	1.23 (0.86–1.77)	0.24	1.03 (0.68–1.54)	0.88
Diabetes	1.34 (0.85–2.09)	0.19	1.58 (0.93–2.67)	0.08
Dyslipidaemia	1.57 (0.79–3.11)	0.18	1.49 (0.69–3.22)	0.30
Elevated fasting blood glucose	1.40 (0.95–2.06)	0.08	1.29 (0.83–1.98)	0.24
Elevated triglycerides	1.11 (0.79–1.57)	0.52	1.17 (0.79–1.74)	0.40
Low high‐density lipoprotein‐cholesterol	2.07 (1.02–4.20)	0.04	2.34 (1.07–5.10)	0.03
High waist circumstance	1.24 (0.88–1.74)	0.20	1.58 (1.06–2.37)	0.02

*Note:* Model 1: the crude effect of endometriosis on every cardio‐metabolic event. Model 2: Mole 1 + adjusted for age, education, smoking, physical activity.

## 4 Discussion

The present study investigated the association between endometriosis and metabolic syndrome. Our findings indicate that women with endometriosis were approximately 2 times more likely to experience metabolic syndrome compared to those without endometriosis. And they are also more likely to experience high waist circumstance and low HDL. We also find no association between EM and other metabolic disorders including diabetes, pre‐HTN and HTN.

In the 21st century, metabolic disorders are considered a challenge for public health [25]; it is a cluster of interrelated risk factors, including abdominal obesity, elevated blood pressure, dyslipidaemia and impaired glucose tolerance or insulin resistance. Inflammation and oxidative stress play a significant role in the development of metabolic comorbidities which lead to metabolic dysfunction [26, 27]. Previous research has suggested a potential link between endometriosis and the subsequent development of other chronic cardiometabolic conditions, though the underlying mechanisms remain unclear [8, 28]. Endometriosis is characterised by inflammation and chronic pain [29] and according to the evidence, there is a link between increased levels of cholesterol and inflammation [30]. It is also proposed that chronic stress can affect the development of EM through metabolic reprogramming [31]. Although many factors contribute to the pathophysiology of metabolic syndrome, several studies show that oxidative stress, in conjunction with chronic inflammatory conditions, is at the core of the development of metabolic diseases [32, 33, 34]. Elevated levels of inflammatory factors such as cytokines, C‐reactive protein and carbohydrate antigen 125, IL‐1, IL‐6, TNF‐α and vascular endothelial growth factor have been found in peritoneal fluid and in the peripheral blood in women with EM [35, 36, 37, 38]. A previous study has reported the presence of abnormalities in the profiles of insulin‐like growth factor (IGF), which have recently been introduced as potential biomarkers for the early diagnosis of metabolic syndrome components, in the peritoneal fluid of women diagnosed with endometriosis [39, 40]. Immune dysregulation also plays role in the development of both endometriosis and metabolic status [41, 42]. It has been shown that external environmental factors including lifestyle are also regarded as drivers of endometriosis [41]; gut microbiota dysbiosis has been reported in animal models and women with EM [43]. Immune dysregulation, inflammatory response and abnormal oestrogen metabolism caused by the imbalance of intestinal homeostasis may stimulate the growth of EM and promote the development and progression of EM [44]. Dysbiosis of the gut microbiota increases the risk of MetS and other chronic diseases, such as obesity, diabetes, dyslipidaemia and hypertension possibly following inflammation, increasing reactive oxygen species and oxidative stress [45, 46, 47, 48, 49, 50]. The major limitation of previous studies is the lack of mechanistic insight into the relationship between endometriosis and associated metabolic disorders. By not exploring these mechanistic pathways, previous studies may overlook critical factors that influence both the development and management of metabolic disorders in this population. Previous studies have also primarily focused on isolated metabolic conditions, failing to provide a comprehensive understanding of the interplay among various metabolic disorders. This narrow focus limits the ability to draw broader conclusions about the overall cardio‐metabolic risk profile in women with endometriosis. Moreover, there is considerable variation in the methodologies used for assessing metabolic disorders across the literature. This inconsistency can lead to difficulties in comparing results and drawing definitive conclusions about the relationship between endometriosis and metabolic health.

Furthermore, women diagnosed with endometriosis have been observed to exhibit lower serum levels of vitamin D compared to healthy controls. This phenomenon may potentially be explained by the loss of the anti‐proliferative actions of vitamin D, which could promote the progression of endometriosis [51]. Additionally, vitamin D has been implicated in the pathogenesis of various metabolic disorders, including cardiovascular disease (CVD) [52].

The increase in risk of hyperlipidaemia and unfavourable lipid profiles including higher levels of LDL‐C, TG and a lower level of HDL‐C have been reported in women with EM [53, 54, 55, 56]. A previous study demonstrated that endometriosis was associated with higher risk of coronary heart disease (CHD) and cardiovascular diseases (CVDs) [57], as it has been well established that hypercholesterolaemia and hypertension are among the strongest risk factors for CHD [58, 59].

Irrespective of the presence of endometriosis, the prevalence of MetS and diabetes among the general female population was found to be 43% and 13.4%, respectively [60, 61]. A systematic review including six cohort studies has demonstrated that endometriosis is associated with an increased risk of various cardiovascular diseases, including ischaemic heart disease and cerebrovascular disease [62]. In a large, prospective cohort study among 116,430 women in the Nurses' Health Study II, laparoscopically confirmed endometriosis was associated with increased risk of CHD [57]. However, our findings demonstrated that odds of pre‐HTN, HTN pre‐DM and diabetes were not significantly different among women with and without EM. In accordance with our study, previous research has demonstrated that women with endometriosis did not exhibit an increased risk of type 2 diabetes overall. However, within subgroups at low inherent risk for type 2 diabetes, endometriosis was associated with a modest increased risk of developing the condition [12]. A cohort study showed increased risk of hypercholesterolaemia and hypertension in women with laparoscopically‐confirmed endometriosis [11]. It is plausible that the chronic inflammatory state associated with endometriosis may impact lipid metabolism through various mechanisms, potentially leading to elevated levels of LDL, which is the primary form of hypercholesterolaemia [63, 64, 65].

Inflammatory responses in the vasculature play key roles in the vascular remodelling process, which may be contributing to blood pressure elevation [66]. Hence, the chronic systemic inflammation in endometriosis may predispose women with endometriosis to a higher risk of hypertension. A review of randomised trials and observational studies showed that non‐steroidal anti‐inflammatory drugs (NSAIDs) use increased the risk of non‐fatal MI with no substantial effect on fatal events [67]. This finding may in part explain that the association between endometriosis and hypercholesterolaemia/hypertension can be partially accounted for by analgesics use. In addition, it is also possible that the use of analgesics was an indicator of chronic pain, which has been linked to an increased risk of cardiovascular diseases [68, 69]. In a study, researchers noticed that women with endometriosis who were not prescribed hormone medications had a higher incidence of hyperlipidaemia than their non‐endometriosis counterparts [53]. It is possible that women with EM in our study have better management of disease that decreases the inflammation and following that development of HTN and diabetes.

The utilisation of a population‐based dataset represents a key strength of the present study, as it enables the findings to be generalised to the broader population. However, the cross‐sectional design of this investigation precludes the establishment of causal relationships between metabolic syndrome and endometriosis. Despite the strengths of the study, several potential limitations must be acknowledged. While the validity of self‐reported endometriosis data has been previously established, the possibility of recall bias should be considered when interpreting the findings. Recall bias may arise when individuals with endometriosis are more likely to accurately report their condition compared to those without the disease, leading to potential misclassification. Such misclassification could influence the observed associations between endometriosis and cardio‐metabolic risk factors, thereby affecting the validity of our conclusions. While we utilised validated questionnaires and corroborated self‐reported endometriosis diagnoses with medical documentation, however, the accuracy of self‐reported data may be influenced by individual perceptions, knowledge and access to healthcare. Furthermore, the exclusion of individuals presenting with mild to moderate chronic pelvic pain, dysmenorrhea or dyspareunia but lacking a definitive diagnosis of endometriosis may limit the generalisability of our findings. There remains a possibility that asymptomatic cases of endometriosis were inadvertently included in our analysis, which could further complicate the interpretation of results.

Additionally, the cross‐sectional nature of the study design limits the ability to determine the temporality of the association between metabolic syndrome and endometriosis. It remains unclear whether metabolic disturbances precede the development of endometriosis or vice versa. Longitudinal studies are needed to elucidate the directionality of this relationship and to identify potential mediating factors. Overall, while these limitations should be considered when evaluating our findings, we believe that our study provides valuable insights into the cardio‐metabolic risk profile of reproductive‐aged women with and without endometriosis. The findings underscore the importance of comprehensive metabolic screening and management in the clinical care of women with endometriosis. Healthcare providers should be vigilant in monitoring metabolic parameters and implementing appropriate lifestyle interventions and/or pharmacological treatments to mitigate the risk of metabolic complications in this patient population. Future research should aim to address these limitations by employing more comprehensive diagnostic criteria and exploring larger, more diverse populations to enhance the robustness and applicability of findings in this area.

## 5 Conclusion

Women with endometriosis are more susceptible to metabolic syndrome, low HDL and high WC than women without endometriosis. The findings underscore the importance of comprehensive metabolic screening and management in the clinical care of women with endometriosis. Healthcare providers should be vigilant in monitoring metabolic parameters and implementing appropriate lifestyle interventions and/or pharmacological treatments to mitigate the risk of metabolic complications in this patient population.

## Author Contributions

All authors conceived of the study, participated in its design and helped to draft the manuscript. Likewise, all authors made suggestions and critical reviews to the initial draft and contributed to its improvement until reaching the final manuscript, which was read and approved by all authors.

## Ethics Statement

The study was approved by the ethical review board of the Research Institute for Endocrine Sciences, Shahid Beheshti University of Medical Sciences, Tehran, Iran. All methods were carried out in accordance with relevant guidelines and regulations with the Declaration of Helsinki.

## Consent

Informed consent was obtained from all participants [IR.SBMU.ENDOCRINE.REC.1403.075].

## Conflicts of Interest

The authors declare no conflicts of interest.

## Data Availability

The datasets used and/or analysed during the current study available from the corresponding author on reasonable request.
